# Takotsubo Cardiomyopathy “Variations on a Theme”

**DOI:** 10.1155/2011/131828

**Published:** 2011-07-17

**Authors:** Kjell Bogaard, Diego A. M. Odekerken, Albertus J. Voogel

**Affiliations:** ^1^Department of Cardiology, Spaarne Ziekenhuis, Spaarnepoort 1, 2134 TM Hoofddorp, The Netherlands; ^2^Department of Cardiology, Onze Lieve Vrouwe Gasthuis, Oosterpark 9, 1091 AC Amsterdam, The Netherlands

## Abstract

Takotsubo Cardiomyopathy (TTC) is a fairly new diagnosis in the cardiologist's repertoire. It can present itself in multiple different forms. We describe three cases of TTC with different etiologies illustrating the broad spectrum of presentations.

## 1. Introduction

Stress-induced cardiomyopathy, Takotsubo, or broken heart syndrome is a fairly new diagnosis in cardiology. Dote et al. were the first to describe the phenomenon of transient apical ballooning induced by emotional stress, with a predisposition for postmenopausal females [1]. Since then several cases have been reported worldwide. 

The “classical” presentation is a postmenopausal woman experiencing acute chest pain after a severe emotional event, with electrocardiographic signs of (transmural) ischemia, transient LV dysfunction, and minimal myocardial enzyme release [2]. In this paper three, nontypical cases of Takotsubo cardiomyopathy (TTC) are described, illustrating the broad spectrum of possible presentations.

**Case 1**

**Figure 1:**
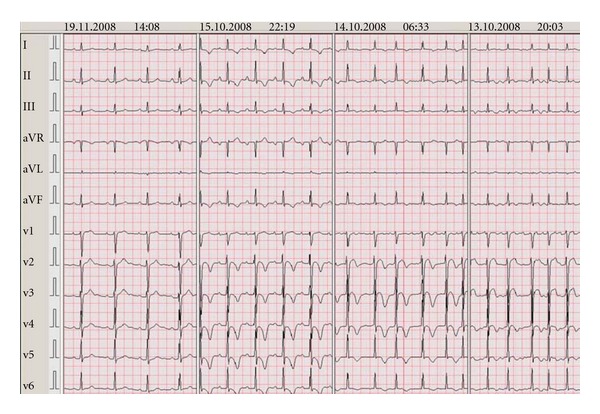
Case  1 (EKGs shown chronologically from right to left).

**Figure 2:**
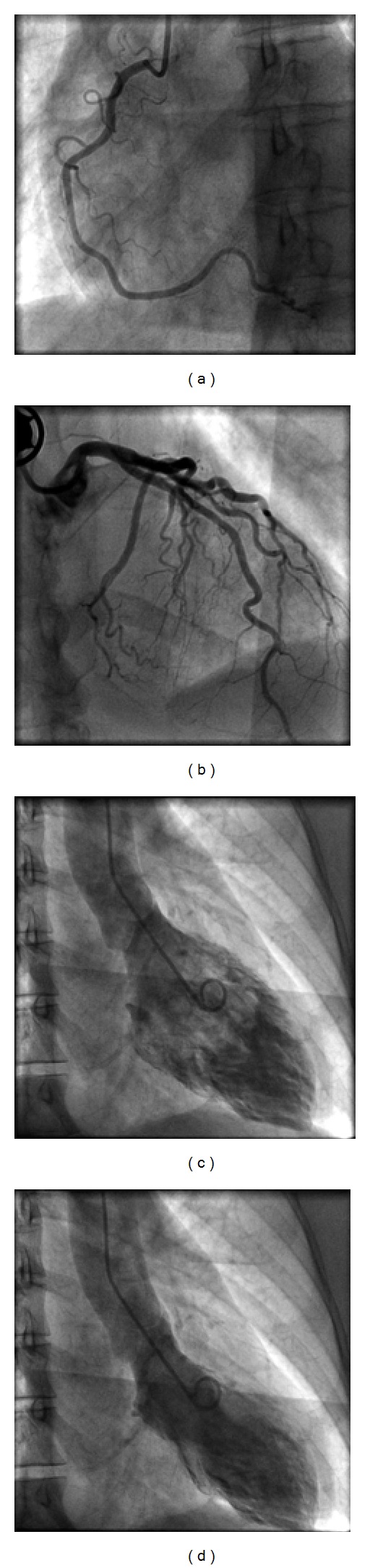
Left ventricular angiogram showing apical ballooning (Case  1).

A 48-year-old, premenopausal female without previous medical history presented with sudden chest pain and palpitations. On the day of the presentation, she encountered no significant physical or emotional stress. The EKG on admission showed atrial fibrillation with rapid ventricular response and ST elevation in V2–V6 (Figure 1). Patient was referred for primary PCI. However, no occlusive coronary artery disease was present. Left ventriculography showed apical akinesia with apical ballooning (Figure 2). Echocardiography was not performed in the acute phase. Laboratory analysis showed not only a maximal Troponin T (TNT) of 0.06 *μ*mol/L, but also signs of hyperthyroidism with a fT4 of 82 *μ*mol/L with a suppression of thyroid-stimulating hormone. The patient was treated with ACEinhibitors, coumadines, and beta-blockade as well as with strumazol. On followup both LV-function (2 weeks) and thyroid function were normalized, and she regained sinus rhythm spontaneously.

**Case 2**

**Figure 3:**
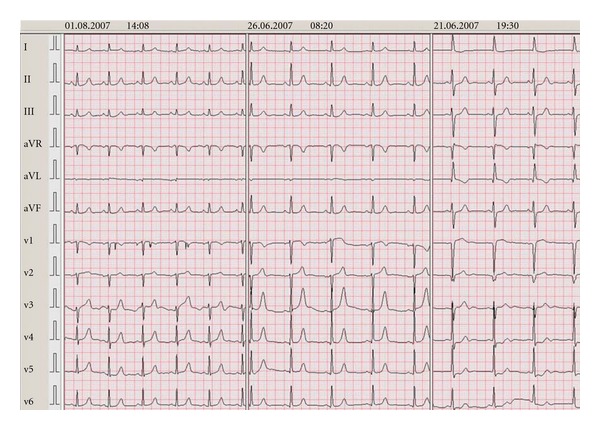
Case  2 (EKGs shown chronologically from right to left).

A 51-year-old, postmenopausal female experienced a “classical” TTC in 2005 following an emotional event, with documented apical ballooning in the absence of coronary artery disease. In 2007, she was readmitted with typical chest pain, again after emotional stress. The patient had been using beta-blockade and statin therapy until the moment of admission. The EKG showed ST depressions (1 mm) in the inferolateral leads, with 1 mm ST elevation in V1-2 (Figure 3). In contrast to 2005, echocardiography showed a hyperdynamic apex with dyskinesia of the basal segments (estimated LVEF 40%). Coronary angiography was again unremarkable and the diagnosis “reverse TTC” was made (See video S1 in supplementary material available online at doi:10.1155/2011/131828). There were no clinical indicators of a pheochromocytoma. Troponin T reached a level of 0.44 *μ*mol/L. Repeated echocardiography after one week showed complete normalization of the LV function.

**Case 3**

**Figure 4:**
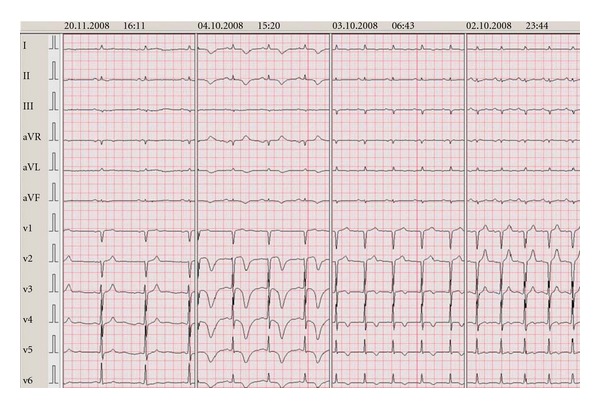
Case  3 (EKGs shown chronologically from right to left).

A 72-year-old male presented with classical chest pain following strenuous exercise. He experienced a transient cardiomyopathy with normal coronary anatomy three years before, which was suspect for myocarditis. Both the clinical picture as well as the imaging studies at that time were non suggestive for a TTC; LVEF was 35% with regional hypokinesia of the inferior wall, both recovered to normal. During admission the patient developed deep negative T-waves in the anterior wall on his EKG and a long QTc (Figure 4). Echocardiography showed akinesia mid-anteroseptal and of the entire apex, with hyperdynamic basal segments. Coronary angiography and ventriculography confirmed the diagnosis of TTC. There were no indicators of a relapse myocarditis. Laboratory results showed a Troponin T of 0.14 *μ*mol/L. Followup showed normalisation of LV function within two days.

## 2. Discussion

We describe three unusual cases of Takotsubo's cardiomyopathy. All three patients showed transient left ventricular dysfunction, in the absence of significant coronary artery disease. The first case showed a TTC in a premenopausal woman, probably provoked by thyrotoxicosis. Case two describes a relapse TTC, of the reverse type, after a previous classical TTC. In case three, a male patient experiences a TTC, without any preceding stressor, three years after having a transient cardiomyopathy of probably a different origin. 

The first two to describe TTC were Iga et al. and Sato et al. [3, 4]. A recent review reported 93% of the TTC cases occurring in postmenopausal women, with a mean age of 67 years [3]. Approximately 2% of all patients presenting with a coronary syndrome suffer from TTC [5]. Most cases are related to extreme emotional (33–45%) and/or physical stress (17–22%) [5]. Several different stressors have been described and summarized by Prasad et al. (Table 1) [6].

Multiple pathophysiological mechanisms have been postulated ranging from acute myocarditis and multivessel coronary artery spasm to increased catecholamine levels. Myocardial biopsy studies have not been able to consequently demonstrate signs of inflammation [6]. A study with 30 TTC patients in Japan, where coronary artery spasm seems to be more prevalent, showed spontaneous multivessel spasm in 3 and inducible spasm in 10 patients [7]. The catecholamine hypothesis, however, seems to be plausible. Increased levels of noradrenalin and catecholamines and microscopic signs of catecholamine toxicity (contraction band necrosis) have been demonstrated in patients suffering from TTC [5, 6]. The findings, however, have not been consistent. Recently Abraham showed that infusion of epinephrine or dobutamine can elicit all the features of stress cardiomyopathy [8].

Until now only few case reports have been published describing hyperthyroidism-related TTC [9]. There are however, several reports describing “vasospastic angina” related to hyperthyroidism [10, 11]. Thyroid hormone is known to increase sensitivity to circulating catecholamines [12], thereby possibly increasing the susceptibility for TTC. 

The “classical” presentation of TTC includes apical akinesia (ballooning); however the second case demonstrates different echocardiographic findings. A review of the literature reveals five different types of segmental dysfunction in TTC (Table 2) [13]. The second case also demonstrated that a large area of wall motion disturbances is not always accompanied by major EKG changes. As far as we know, there is no real explanation for this observation. Recently Dib et al. Showed the lack of correlation between EKG abnormalities and the extent of wall motion abnormalities [14].

A recent large study described a recurrence rate of 11.4% in 4 years [15]. Two of our patients experienced a transient cardiomyopathy before, differing from the recent TTC presentation. The prognosis, however, seemed to be quite good since the four-year survival was not different from that in an age-matched and gender-matched population. 

Concluding Takotsubo Cardiomyopathy is a fairly new diagnosis in the cardiologist's repertoire. It can present itself in multiple forms with or without the typical stressors; hyperthyroidism appears to be one of the possible stressors and should be evaluated routinely. Once a patient suffered from a TTC, a relapse is a realistic possibility.

## Supplementary Material

Transthoracic echocardiography showing a hyperdynamic apex with dyskinesia of the basal segments on the left side and complete normalisation of the wall motions on the right side after 1 week.

## Figures and Tables

**Table 1:** Stressors reported to trigger TTC [6].

(i) Emotional stress
(ii) Death or severe illness or injury of a family member, friend, or pet
(iii) Receiving bad news—diagnosis of a major illness, daughter's divorce,
(iv) Spouse leaving for war
(vi) Severe argument
(vii) Public speaking
(viii) Involvement with legal proceedings
(ix) Financial loss—business, gambling
(x) Car accident
(xi) Surprise party
(xii) Move to a new residence

(xiii) Physical stress
(xiv) Non-cardiac surgery or procedure—cholecystectomy, hysterectomy
(xv) Severe illness—asthma or chronic obstructive airway exacerbation,
(xvi) connective tissue disorders, acute cholecystitis, pseudomembranous colitis
(xvii) Severe pain—fracture, renal colic, pneumothorax, pulmonary embolism
(xviii) Recovering from general anesthesia
(xix) Cocaine use
(xx) Opiate withdrawal
(xxi) Stress test—dobutamine stress echo, exercise sestamibi
(xxii) Thyrotoxicosis

**Table 2:** Transient venticular cardiomyopathies [13].

Type 1	Apical ballooning Takotsubo cardiomyopathy
Type 2	Mid-ventricular ballooning
Type 3	Apical sparing cardiomyopathy
Type 4	Basal ballooning
Type 5	Other segmental involvement
